# The Germ of Small-Pox and Vaccinia

**Published:** 1901-03-09

**Authors:** 


### THE GERM OF SMALL-POX AND VACCINIA.

It has always seemed odd that the bacteriologist has found it far easier to prove the microbic origin, and thus, presumedly, the infective nature of a series of diseases which used not to be considered infectious at all, than to lay his hands upon the living germs which, as we cannot hesitate to believe, must be the causes of the various eruptive fevers. Thus, to take extreme examples, while tuberculosis was dragged into the category of the infections on the strength of the discovery of the *Bacillus tuberculosis*, small-pox, the very type of an infectious disorder, declined to display the germ on which its infectivity depended. Some time ago, Dr. Monckton Copeman discovered a microbe in vaccinia, but even he failed to cultivate it, so that its claims to be regarded as the cause of the disease remained somewhat uncertain. Now two papers [1] on the subject are published simultaneously, one by Dr. Copeman, and the other by Dr. M. Funck, of Brussels. Dr. Copeman says that at last, by enclosing a small quantity of beef broth inoculated with a minute trace of vaccine lymph, free from extraneous microbes, in capsules, and placing these capsules within the peritoneal cavity of rabbits and dogs, he has been able to obtain a culture, numerous zooglea masses being visible, made up of bodies resembling spores. "Apparently they represent the resting stage of the specific microbe." Dr. Funck takes quite another line. He holds that vaccinia is not a microbic disease, but is caused by a protozoon, easily found in all vaccine pustules and in all active vaccine; that the inoculation of this protozoon in a sterile emulsion reproduces in susceptible animals all the classical symptoms of vaccinia, and renders them refractory to subsequent inoculations with vaccine; that the variolous pustule contains a protozoon morphologically similar to that in the vaccine; and from these facts he argues that vaccinia is but an attenuated form of variola, which, of course, is what is already pretty widely accepted, although on other grounds. No doubt in time Dr. Funck will publish a more complete account of his researches; meanwhile it may be well to point out that he apparently accepts the fact that lymph which is, as he describes it, "sterile," will still produce vaccinia with characteristic pustules, as proof that the active cause cannot be a microbe, and must therefore be a protozoon. Sterility is, however, a merely negative term. A thing is sterile until it is shown to be capable of growth, and the tricks and dodges to which Dr. Copeman had to resort before he could get a culture show how little dependence is to be placed on negative results. The matter is of considerable interest, but Dr. Funck's hypothesis is far from proven.

[1] Brit. Med. Jour., Feb. 23.

